# Differing Roles of Hyaluronan Molecular Weight on Cancer Cell Behavior and Chemotherapy Resistance

**DOI:** 10.3390/cancers10120482

**Published:** 2018-12-03

**Authors:** Zoe K. Price, Noor A. Lokman, Carmela Ricciardelli

**Affiliations:** Discipline of Obstetrics and Gynaecology, Adelaide Medical School, Robinson Research Institute, University of Adelaide, South Australia 5000, Australia; zoe.price@adelaide.edu.au (Z.K.P.); noor.lokman@adelaide.edu.au (N.A.L.)

**Keywords:** hyaluronan, cancer, molecular weight, therapy resistance, cancer stem cells, oligosaccharides, tyrosine kinase

## Abstract

Hyaluronan (HA), a glycosaminoglycan located in the extracellular matrix, is important in embryo development, inflammation, wound healing and cancer. There is an extensive body of research demonstrating the role of HA in all stages of cancer, from initiation to relapse and therapy resistance. HA interacts with multiple cell surface receptors, including CD44, receptor for hyaluronan mediated motility (RHAMM) and intracellular signaling pathways, including receptor tyrosine kinase pathways, to promote the survival and proliferation of cancer cells. Additionally, HA promotes the formation of cancer stem cell (CSC) populations, which are hypothesized to be responsible for the initiation of tumors and therapy resistance. Recent studies have identified that the molecular weight of HA plays differing roles on both normal and cancer cell behavior. This review explores the role of HA in cancer progression and therapy resistance and how its molecular weight is important in regulating CSC populations, epithelial to mesenchymal transition (EMT), ATP binding cassette (ABC) transporter expression and receptor tyrosine kinase pathways.

## 1. Introduction

In 2012, there were an estimated 14.1 million new cancer cases diagnosed and 8.2 million cancer related deaths globally [1]. These statistics signify the massive burden of cancer on society, affecting both developed and underdeveloped regions. Therapy resistance is a major problem impacting patient survival and accounts for treatment failure in up to 90% of metastatic cancers [2]. Tumor cells have developed multiple mechanisms for overcoming cancer therapies, in particular up-regulation of ATP binding cassette (ABC) transporters responsible for efflux of cancer therapies and activation of pro-survival and anti-apoptotic pathways [3]. There is also the existence of tumor cells with stem cell phenotypes including enhanced proliferation, therapy resistance and the ability to differentiate [4]. These cells, referred to as cancer stem cells (CSC) are believed to survive initial chemotherapy treatment, repopulate relapse tumors and promote metastasis [5]. Due to the massive burden of chemotherapy resistance on patient survival, there has been a great deal of research towards identification of novel, therapeutic targets for overcoming chemoresistance. The sugar molecule hyaluronan (HA), located in the extracellular matrix (ECM), has been linked to cancer initiation, progression, metastasis, relapse and therapy resistance [6,7,8]. Methods for identifying the biological role of HA include overexpression, inhibition and knockdown of HA synthesis and processing enzymes and the addition of exogenous HA. Exogenous HA has allowed the identification of the importance of HA molecular weight in mediating biological effects. This review discusses the role of HA in cancer progression and therapy resistance and explores how its molecular weight is important in mediating its functional role in both normal and cancer cells.

## 2. Hyaluronan

### 2.1. HA Synthesis, Degradation and Conformation

HA, a major component of the ECM, was first discovered in 1934 by John W. Palmer and Karl Meyer who isolated a high molecular weight polysaccharide from bovine vitreous humor [9]. HA interacts with a range of cell surface receptors, including cancer stem cell marker CD44, receptor for hyaluronan-mediated motility (RHAMM), toll like receptors 2 and 4 (TLR2, TLR4), hyaluronan-binding protein 1 (HABP1), hyaluronan receptor for endocytosis (HARE) and lymphatic vessel endothelial receptor for hyaluronan 1 (LYVE1). In normal tissues, HA can promote cell proliferation, migration and adhesion in processes including embryogenesis, tissue regeneration and wound healing [10]. It is produced on the inner side of the plasma membrane by HA synthases (HAS1-3) which polymerise disaccharides of N-acetyl-D-glucosamine and D-glucuronic acid by a β-(1-3)-glucuronidic bond [11]. HAS2 produces the largest polymers of HA at > 2 × 10^6^ Da, HAS1 with a range from 2 × 10^5^ to 2 × 10^6^ Da and HAS3 with a range of 1 × 10^5^ to 1 × 10^6^ Da [12]. HA polymers can also be catabolized endogenously by hyaluronidases (HYAL1-3) which hydrolyse the β-(1-4)-hexosaminidic bond, processing polymers of high molecular weight HA (HMW-HA) of > 1 × 10^6^ Da, to low molecular weight HA (LMW-HA) of 0.8 to 8 × 10^5^ Da and HA oligosaccharides (oHA) of less than 6 × 10^3^ Da [13]. Specifically, HYAL2 processes HMW-HA to polymers of approximately 20 kDa which are further processed by HYAL1 into oHA [14]. HA polymers are also processed in oxidative reactions by reactive oxygen species and free radicals which generate shortened HA fragments with oxidized termini (reviewed in [15]). The presence of a mutant HAS2 and decreased Hyal activity has been linked to tumor resistance in the naked mole rat [16,17]. Two 100% conserved asparagines are replaced by serines, in the active site of HAS2 producing a very high molecular weight HA (vHMW-HA) of 6–12 MDa [17,18].

The use of synthetic membranes and sensors has allowed assessment of effects of molecular weight of HA on its binding affinities and receptor clustering. Increasing HA molecular weight up to 1000 kDa was associated with an increased CD44 binding affinity and increased CD44 clustering [19,20]. HA polymers have multiple binding sites whereas oHA of 6 to 10-mers occupy one HA binding domain (HABD) and oHA of 18 to 22 mers occupy two HABD on CD44 [20,21]. Additionally, whilst native HA can promote CD44 clustering, oHA can reduce CD44 clustering by competitively disrupting native HA binding [21,22]. A recent study by Weigel and Baggenstoss demonstrated HA polymers of 150–250 kDa exist in a conversion state between rod like and coil conformation, whilst HA polymers below 150kDa exhibit rod-like conformation and polymers above 250 kDa exhibit coil-like conformations [23]. The different interactions with CD44, binding affinities and receptor clustering and different conformations of HA may help explain the varying effects of different molecular weights of HA in normal and cancer cell biology.

### 2.2. HA in the Tumor Microenvironment

Malignant transformation of cells is not solely dependent on genetic mutations but also requires interactions with the tumor microenvironment which is composed of stromal cells including fibroblasts, myofibroblasts, also known as cancer associated fibroblasts (CAFs), and a variety of immune cells including tumor associated macrophages which are localized within the ECM [24,25]. Prior to malignant transformation, the stroma becomes more fibrotic and is characterized by the overproduction of ECM molecules including HA [25]. Increasing HA production by fibroblasts is capable of promoting their activation to CAFs [25,26]. HA is a major component within the ECM, it binds to hyaladherins such as versican or receptor CD44, forming a complex matrix which not only provides signals to various cells but also retains water, increasing interstitial fluid pressure, aiding metastasis and therapy resistance [24].

**Figure 1:**
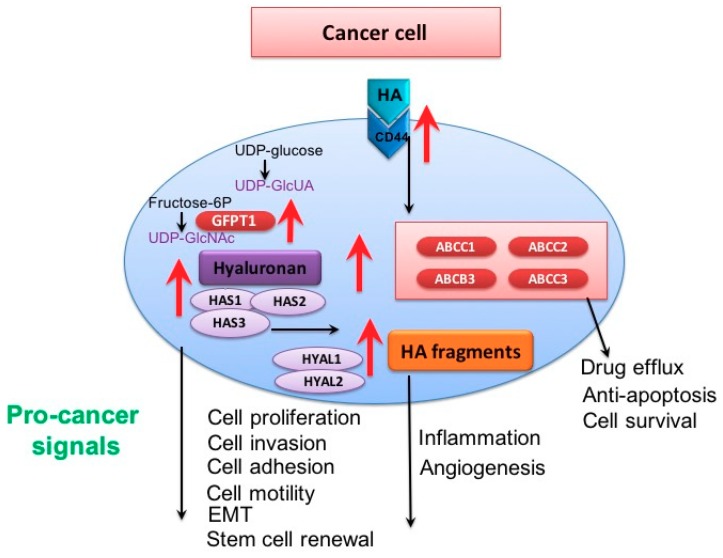
Cancer cells produce increased levels of endogenous hyaluronan (HA) which is exported from the cells and interacts with receptor CD44 to promote expression of ATP binding cassette (ABC) transporters and subsequent drug efflux and cell survival. HA polymers are also digested by hyaluronidases producing HA fragments which promote inflammation and angiogenesis. HA polymers activate signaling pathways which send a range of pro-cancer signals promoting cell proliferation, invasion, adhesion, motility, epithelial to mesenchymal transition (EMT) and stem cell renewal. UDP: uridine diphosphate; HAS: hyaluronan synthase.

HA plays an important role in cancer initiation, proliferation, metastasis and progression (recently reviewed in [25,27,28]). Pro-tumorigenic behavior mediated by HA include cancer cell proliferation, motility, invasion, adhesion, epithelial mesenchymal transition (EMT) and cancer stem cell (CSC) activation (Figure 1). HA expression, HAS and HYAL enzymes and cellular HA levels have also been associated with patient outcome in a wide range of cancers [29]. High levels of stromal HA have been associated with relapse and reduced overall survival in breast and ovarian cancer tissues [30,31] and associated with malignant and clinicopathological features in prostate cancer [32]. The increased HA production in stromal cells is stimulated by tumor cells [33,34]. Expression of hyaluronidases and HA synthases have also been linked with reduced prognosis, therapy response and tumor pathology in a variety of tumors including prostate, ovarian, head and neck and breast carcinoma [35,36,37,38,39].

## 3. Role of Molecular Weight of Hyaluronan in Normal Biology

There is growing evidence of the importance of molecular weight of HA for its normal biological function including immune regulation, angiogenesis, wound healing and development. Studies highlighting the importance of HA molecular weight on normal cells are summarized in Table 1. Initial studies demonstrated HA molecular weight was important in inflammation, LMW-HA was pro-inflammatory and HMW-HA anti-inflammatory [10,27,40]. In mouse knee chondrocytes, LMW-HA 50 kDa promoted expression of inflammatory cytokines and inflammatory response receptors including toll like receptor 4 (TLR4), HMW-HA 5000 kDa decreased TLR4 expression whilst MMW-HA 1000 kDa had no effect [41]. Another study found HMW-HA 1600 kDa and MMW-HA 900 kDa decreased the stimulated inflammatory response in squamous nasal epithelial cells (RPMI 2560) where as LMW-HA 370kDa had no effect [42]. These findings suggested HA below 370 kDa may be responsible for activating an inflammatory response. Platelets isolated from human plasma were found to contain HYAL2 hyaluronidase which can cleave HA into a range of smaller fragments that promoted the production of pro-inflammatory cytokines by monocytes [43]. From these observations Motte et al. proposed an inflammatory feedback loop where endothelial cells respond to inflammatory stimuli, increasing production of HA. The HA is then recognised by platelets and processed into LMW fragments via HYAL2 which promote monocyte activation [43]. There is conflicting evidence for the initial pro-inflammatory effects of LMW-HA [44]. Neither LMW-HA (11-250 kDa) or HMW-HA (970 kDa) stimulated nitric oxide or tumor necrosis factor alpha (TNF-α) expression in mouse macrophage cell lines RAW 264.7 and MHS [45]. 3 kDa purified HA was also unable to stimulate IL-6 production in primary mesangial cells but treatment with lipopolysaccharide (LPS) contaminated Hyal stimulated IL-6 secretion [46]. This suggested the possibility the pro-inflammatory effects of LMW-HA by biological sources of HA and Hyal were due to endotoxins rather than the LMW-HA [44]. Analysis of HA from *Streptococcus zooepidemicus*, rooster comb, bovine vitreous and umbilical cord found umbilical cord and bovine vitreous HA to have high protein nucleic acid contamination [47] and endotoxin contamination [44]. Umbilical cord HA and Hyal (bovine and *Streptococcus hyaluronlyticus*) preparations with endotoxin contamination stimulated TNF-α and IL-12 production in CFS-2 bone marrow derived macrophages and dendritic cells (BMDM and BMDC) whereas purified HA samples of varying sizes (HA 28–1680 KDa, Life Core) and bovine HA with low endotoxin levels did not elicit a pro-inflammatory response [44]. This was inconsistent with another study utilising purified HA samples (5, 60, 800 and 3000 kDa, Lifecore), where 5 kDa and 60 kDa but not 3000 kDa HA stimulated TNF-α and nitrous oxide secretion in murine J744A.1 ATTC and TIB-67 macrophages [48]. This variation could possibly be due to differential responses between primary macrophages and cell lines. Cell presentation could also affect the response to HA as seen in BALB/c macrophages which only responded to LMW-HA in adherent conditions [49]. Receptor interactions could also be important as LMW-HA has been shown to mediate inflammation through TLR-2, TLR-4, CD44 and HARE [41,49,50,51]. A novel finding for receptor complexes was also demonstrated for a CD44 and TLR-4 complex, where LMW-HA (200kDa) inhibited lung inflammation [52]. Together these findings highlight the possibility the effects of HA in inflammation are not solely dependent on molecular weight. HA purity, cell type, receptor expression and cellular context all need to be considered when determining the role of HA molecular weight. Interestingly, a study in peripheral blood mononuclear cells demonstrated an alternate role for HMW-HA in the inflammatory response, finding 2000 kDa HA and not LMW-HA 80–800 kDa, promoted differentiation of monocytes to fibroblast-like cells called fibrocytes [13]. The study found that LMW-HA inhibited the ability of HMW-HA to promote production of anti-inflammatory cytokines, interleukin-4 (IL-4) and interleukin-13 (IL-13) [13].

Molecular weight of HA is also important in angiogenesis and wound repair. oHA (2–10 disaccharides) promoted the proliferation of bovine aortic endothelial cells (BAEC) and cell migration in wound assays via mitogen activated protein kinase (MAPK) signaling whereas HA polymers (unknown and 500–1000 kDa) had opposing effects [53,54,55]. oHA also activated MAPK in porcupine vascular endothelial cells promoting proliferation and migration [56]. The effects of oHA (2–10 disaccharides) have also been observed in vivo, where oHA increased capillary density and wound healing in Sprague-Dawley rats [57]. The size of oHA can also mediate their function. A study by Tolg et al. analysed oHA of 4mer, 6mer, 8mer and 10mer and 40kDa and 500kDa native HA on wound healing in vitro (rat dermal fibroblasts) and in vivo (sprague-dawley rats) and found only 6mer and 8mer oHA promoted wound healing in both settings [58]. NIH-3T3 mouse fibroblasts responded to HMW-HA 980 kDa and not 132 kDa, 31 kDa or 2.3 kDa HA, promoting NFκβ activation and subsequent Snail2 expression and invasion [59]. 2100 kDa HA also promoted smooth muscle cell migration [60].

Molecular weight of HA is also crucial during embryogenesis. HMW-HA and MMW-HA (unspecified molecular weight) activate the phosphatidylinositol 3-kinase (PI3K) and MAPK pathways in primary trophoblasts to promote cell proliferation, invasion and survival, whereas LMW-HA (unspecified weight) has no effect [61]. LMW-HA is important in gland morphogenesis [62,63]. HA fragments between 7–21 kDa could stimulate mammary gland branching whilst 500 kDa HA inhibited branching [62]. A similar effect of LMW-HA has been observed on ureteric gland branching in the kidney, with lower molecular weight of HA (17 kDa and 6.5 kDa) stimulating branching morphogenesis and high molecular weight HA (234.4 kDa and 132.3 kDa) having inhibitory effects [63].

## 4. Role of Molecular Weight of Hyaluronan in Cancer Biology

**Figure 2:**
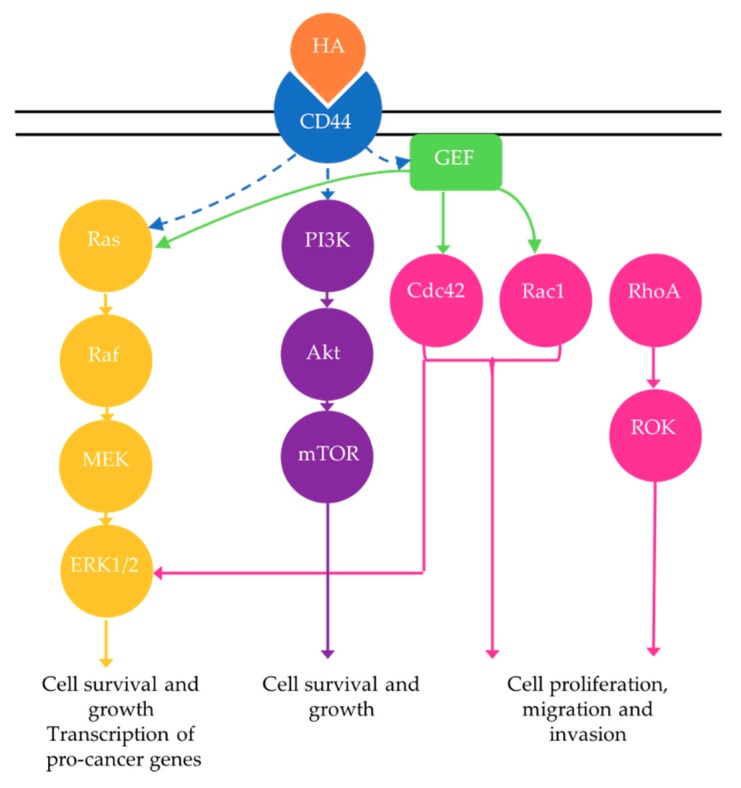
HA interacts with cell surface receptor CD44 which indirectly activates Rho, MAPK and PI3K signaling cascades to promote cell survival, growth, proliferation, migration and invasion and transcription of pro-cancer genes.

In addition to a role of molecular weight of HA in normal biology, there is increasing evidence that HA molecular weight is also important in cancer cell biology and involved in regulating CSC populations, EMT, ABC transporter expression (Figure 1) and the regulation of receptor tyrosine kinase pathways including PI3K, RhoGTPase and MAPK (Figure 2). Molecular weight of HA may also be important as a diagnostic marker in cancer as LMW-HA (< 50 kDa) correlated with metastasis in breast cancer [64]. Multiple studies have utilized exogenous HA of known molecular weights demonstrating effects of specific molecular weights in these cancer cell pathways (Table 2).

### 4.1. Cancer Stem Cells, Hyaluronan and Therapy Resistance

The initial hypothesis proposed for the initiation of cancer was clonal expansion, where over time cells gradually accumulate mutations passed down through proliferation which can promote the malignant transformation of cells [79]. A more recent hypothesis is the CSC hypothesis which postulates a small population of cells with stem-like features generate tumors via a hierarchy [4]. The presence of tumors that have genetic and phenotypic heterogeneity but lack the hierarchy of the CSC model has also suggested both mechanisms may be important in tumor initiation [80]. HA has been shown to promote the formation of CSC populations in breast cancer [81] and glioblastoma [82]. Additionally, HA activates genes associated with stemness in embryogenesis and interacts with CSCs to enhance stemness and therapy resistance [83].

CSCs have the ability to promote tumor formation from low cell counts with the same heterogeneity as the initial tumor [84]. CSC are defined by a range of markers. Ovarian CSCs are defined by aldehyde dehydrogenase (ALDH), CD44, CD133, CD117, ABCG2, epithelial cell adhesion molecule (EPCAM) and CD24 [85,86]. Head and neck cancer stem cells are defined by markers CD44, CD133, ALDH and c-MET, whilst breast cancer cells are defined by markers CD24, CD44, CD133, EPCAM, PIWIL2 and ALDH [87,88]. Different combinations of these stem cell markers are associated with decreased progression free survival and overall survival for ovarian, head and neck and breast cancer [89,90,91,92,93,94,95]. Three transcription factors, NANOG, SOX2 and OCT4 facilitate the expression of genes involved in induction and maintenance of pluripotency in CSCs [96,97]. Overexpression of NANOG, SOX2 and OCT4 have been linked to therapy resistance, reduced overall survival and increased progression in ovarian cancer, head and neck, breast, renal and rectal cancer patients [91,98,99,100,101,102,103,104].

ALDH positive SKOV-3 and Hey1 ovarian CSCs and primary ovarian tumor cells positive for ALDH and CD133 have enhanced resistance to therapies and exhibit enhanced tumor growth in vivo [105]. In addition, chemotherapy treatment enhances populations of CSCs. Treatment of high grade serous ovarian cancer cells, OVCA433 and ovarian mesenchymal cells Hey, with cisplatin and paclitaxel enhanced cell populations with stem cell phenotypes, promoted in vitro sphere formation, expression of CSC genes (*CD44, EPCAM, CD117, OCT4A* and NANOG) and in vivo metastasis [106]. Enrichment of CSCs following chemotherapy treatment has also been observed in PLC/RAF/5, Huh7 and HepG2 hepatocellular carcinoma cells [107,108].

A study by Bourguignon et al. in ovarian cancer (SKOV-3) and breast cancer (MCF-7) cells, demonstrated 500 kDa HA interacts with CD44 to promote formation of a complex between CD44, Nanog and signal transducer and activator of transcription 3 (STAT-3) which promotes *SOX2, REX1* and *MDR1* expression, cell growth and resistance to doxorubicin and paclitaxel [67]. Further research in MCF-7 cells, demonstrated activation of Nanog by 500 kDa HA promoted cell survival and therapy resistance via upregulation of *miR-21* and downregulation of tumor suppressor programmed cell death 4 (PDCD4) [109]. Formation of the CD44-Nanog-STAT-3 complex by 500 kDa HA and subsequent upregulation of miR-21 and downregulation of PDCD4 has also been demonstrated in head and neck cancer cells (HSC-3) [110]. In a CD44v3^high^ALDH1^high^ population isolated from HSC-3 cells, the interaction of 500kDa HA with CD44v3 promoted the formation of the Oct4-Sox2-Nanog transcription complex and expression of *miR-302* involved in maintaining stemness [111]. Shiina et al. demonstrated molecular weight of HA was important in promoting and maintaining stemness of CSCs, finding 200 kDa HA significantly promoted expression of cancer stem cell genes, sphere and clone formation and cisplatin resistance in ALDH^high^ CD44v3^high^ HSC-3 cells compared to 5, 20 and 700 kDa HA [75]. These studies suggest a possible molecular weight range of HA 200–500 kDa in promoting stemness in cancer cells, however this needs to be confirmed in other cancer models.

Although still controversial, a theory into the initiation of CSCs is via EMT [112]. There is clinical evidence of a link between EMT and CSCs, a particular study in breast cancer patients demonstrated a correlation between expression of EMT transcription factors *Snail* and *Zeb1* and the presence of circulating tumor cells with CSC phenotypes CD326^−^CD45^−^ and ALDH^+^CD133^+^ [113]. Clinical evidence between CSC populations and expression of EMT genes has also been observed in colon, pancreatic and head and neck cancers [114,115,116,117]. The mechanisms which connect CSC with EMT are still yet to be elucidated. HA has been shown to effect EMT in cancer cells (Figure 1) [81]. HAS2 is important during mouse embryo development, due to promotion of EMT [29]. HAS2 was necessary for TGFβ stimulated EMT in normal mouse mammary epithelial cells [118]. Overexpression of HAS2 promoted EMT in breast cancer cells (MCF-10) and Madin-Darby canine kidney epithelial cells [119]. An in vivo study of breast cancer by Chanmee et al. demonstrated overproduction of endogenous HA by HAS2 increased EMT through up regulation of Snail and Twist and down regulation of E-cadherin [81]. In addition, there was a significant increase in a side population of primary breast CTC CD44^high^/CD24^low^ and sphere formation [81]. Overproduction of HA via HAS1 in MCF-10 breast cancer cells also promoted EMT [120]. Zhao et al. demonstrated that different molecular weights of HA can affect EMT [72]. 35kDa HA in an alginate matrix downregulated E-cadherin expression and upregulated vimentin to promote cell invasion, migration and spheroid formation whereas 117 kDa had opposing effects in 4T-1 and SKBR3 breast cancer cells [72]. 3–5 kDa and not 500–1000 kDa HA promoted inflammation and cell invasion in MDA-MB-231 cells via CD44 and TLR receptors [71]. Cell invasion in breast cancer cells is also increased by 500 kDa and 1000 kDa HA [68,69,70]. The variation in HA molecular weight effects on cell invasion is likely due to both receptor presentation and interactions as CD44 often forms complexes with other receptors to stimulate signals. Additional studies using a range of HA molecular weight in a range of cancers are required to determine the importance of HA molecular weight in mediating EMT.

### 4.2. Hyaluronan, ABC Transporters and Therapy Resistance

Multidrug resistance (MDR) is a common occurrence in cancer, where cancer cells develop resistance to multiple, unrelated cancer therapies. Tumors develop a wide array of mechanisms for overcoming drug therapies including increased efflux and decreased uptake of chemotherapy drugs, activation of DNA repair mechanisms, detoxifying systems and inhibiting apoptotic pathways [3,121]. ABC transporters are transmembrane glycoproteins involved in ATP-dependent transport of xenobiotics, lipids and metabolic products across plasma and intracellular membranes [122,123]. There are 48 known human ABC transporters, 20 of which have been shown to export anti-cancer drugs from cancer cells [123]. The most well characterized ABC transporters for MDR are multidrug resistance protein 1 (MDR1) also known as p-glycoprotein or ABCB1, multidrug resistance-associated protein 1 (MRP1) or ABCC1 and breast cancer resistance protein (BCRP) or ABCG2 [124]. MDR1 confers resistance to doxorubicin, paclitaxel, topotecan and docetaxel but not platinum based therapies such as cisplatin [123]. ABCC1 confers resistance to doxorubicin, etoposide and vincristine and ABCG2 confers resistance to doxorubicin and topotecan [125,126].

HA can regulate expression of several ABC transporters in cancer (Figure 1). Overexpression of HAS2 in breast cancer stimulated ABCB1/MDR1 expression through the PI3K pathway increasing resistance to doxorubicin [127]. 500 kDa HA also stimulated MDR1 expression via CD44 in breast cancer (MCF-7 cells) inducing resistance to doxorubicin, paclitaxel and etoposide [67,69,109]. Ricciardelli et al. demonstrated umbilical cord HA significantly increased the expression of *ABCB3, ABCC2* and *ABCC3* in OVCAR-5 and OV90 and *ABCC1* (MDR1) in OVCAR-5 ovarian cancer cells [8]. Carboplatin treatment increased *ABCC2* and HA in OVCAR-5 cells [8]. Increased expression of ABC transporters following HA treatment was not observed in OVCAR-3 cells, which lacked CD44 but was reduced by oHA in CD44 expressing cell lines, demonstrating that ABC transporter expression involves CD44 [8]. HA of unspecified molecular weight also promoted expression of *ABCC2* in non-small cell lung cancer cells [128]. These studies indicate a role for HA in influencing ABC transporter expression and subsequent MDR in cancers. However, it is not known whether the effect on ABC transporter expression is dependent on HA molecular weight and therefore warrants further investigation.

oHA can abrogate chemoresistance and antagonize CD44 interactions with HA polymers [8]. They have also been shown to inhibit ABCB1 activity and the PI3K pathway in doxorubicin-resistant, vincristine-resistant and doxorubicin-sensitive lymphoma cells, increasing sensitivity to vincristine [129]. oHA down-regulated *ABCG2/BCRP* expression in C6/lacZ7 glioma cells [130]. In ovarian cancer, oHA disrupted the localization of ABCB1 with CD44 and inhibited drug efflux function [131]. Additionally, in OVCAR-5 and OV90 cells, oHA decreased expression of *ABCB3, ABCC2* and *ABCC3*, increasing sensitivity to carboplatin [8]. Overall, oHA has been shown to reduce ABC transporter transcription and function, increasing sensitivity of tumors to therapies. However further studies are required to determine whether these observations only relate to oHA.

### 4.3. Hyaluronan, Receptor Tyrosine Kinase Pathways and Therapy Resistance 

HA receptors including CD44 activate downstream receptor tyrosine kinase pathways, including PI3K, which attracts guanine exchange factors (GEF) and can indirectly activate downstream GTPases including Rho GTPases and Ras GTPases in the Rho and MAPK signaling pathways (Figure 2) [60,74].

#### 4.3.1. Rho GTPase Signaling

Rho GTPases are small GTP binding proteins involved in signal transduction mediating cell motility [132]. They undergo conformation activation by GEF which promote the dissociation of GDP in exchange for GTP, allowing interactions with target proteins [133]. There are 16 members in the Rho GTPase family, the most widely studied are Rho, Cdc42 and Rac [134]. Rho GTPase signaling promoted motility of cancer cells via actin polymerisation which can effect cell stiffness and mediate MDR [134]. In ovarian cancer, activation of RhoGTPases increased cell stiffness via actin polymeration which subsequently conferred resistance to cisplatin treatment which was reversed by Rho inhibition [135,136]. Activation of Rho GTPases in response to HA has been demonstrated in breast, head and neck and ovarian cancer. In particular, 500 kDa HA via CD44 activated downstream RhoC and Rho associated kinase (ROK) in HSC-3 head and neck cancer cells and promoted cell growth, invasion, migration and resistance to cisplatin [74,76]. 500 kDa and 1000 kDa HA via CD44 indirectly activated Rho pathways in MDA-MB-231 breast and SKOV-3 ovarian cancer cells promoting cell growth and migration [65,66,68,70]. 500kDa HA activated Cdc42 and 1000 kDa HA activated Rac in SKOV-3 ovarian cancer cells [65,66]. Stimulation of RhoGTPase by 500 kDa and 1000 kDa HA has clearly been demonstrated, however the importance of HA molecular weight in the activation of RhoGTPase pathway in cancer cells has not been investigated.

#### 4.3.2. Phosphoinositide 3-Kinase (PI3K) Pathway

The PI3K pathway is involved in cancer cell growth, proliferation, glucose metabolism, invasion, metastasis, angiogenesis and survival [137]. There are three classes of PI3K, with class I regulating cell growth [138]. Class I has two subclasses, class IA which are activated by receptor tyrosine kinases and class IB which are activated by G-protein coupled receptors. The main effector of the PI3K pathway is protein kinase B or Akt, a serine threonine kinase whose acts on the mTOR complex [139]. Activation of the PI3K pathway can both suppress cell death and promote cell survival. Akt suppressed apoptosis via phosphorylation of pro-apoptotic protein BAD [140] and pro-apoptotic transcription factors FoxO1 [141], FoxO3 [142] and FoxO4 [143]. Akt promoted cell survival via phosphorylation of cell survival proteins including Bcl-2, Bcl-x_L_, MCL-1, A1 and BAG-1 [139]. The PI3K pathway was identified as the most frequently altered pro-cancer pathway in ovarian cancer [144]. Activation of PI3K, Akt and mTOR in ovarian cancer promoted cell proliferation and invasion [145]. Additionally, activation of the PI3K pathway has been linked to therapy resistance in gastric, ovarian, uterine and colon cancer [146,147,148,149]. 500 kDa and 1000 kDa HA via CD44 activated downstream PI3K in head and neck cancer and breast cancer respectively promoting cell proliferation, motility, invasion and therapy resistance [68,74]. Contradictory to this, LMW-HA (~200 kDa) and not HMW-HA (~1600 kDa) activated PI3K pathway in JEG-3 choriocarcinoma cells via RHAMM, promoting cell migration [77]. These studies suggest that different cell types can respond to different HA molecular weights via different receptors. Lompardia et al. demonstrated HMW-HA (1500–1800 kDa) promoted cell growth in K562 leukaemia cells (sensitive to vincristine) via CD44, MAPK and PI3K signaling pathway whereas in Kv562 (resistant to vincristine) HMW-HA activated only the PI3K pathway via RHAMM [78]. 1500–1800 kDa HA also activated the PI3K pathway in lymphoma cells [129,150]. oHA inhibited HA-CD44 interactions demonstrated by inhibition of cell growth in only K562 cells [78]. Additionally, oHA inhibited the activation of the PI3K pathway in lymphoma and colon carcinomas [129,151]. Although specific studies have explored the role of HA in activation of the PI3K pathway there is conflicting evidence between cell types. A wider analysis is needed to clarify the role of HA molecular weight in activating the PI3K pathway in cancer cells.

#### 4.3.3. MAPK Pathway

The MAPK pathway is a signal transduction pathway involving RAS GTPase which activates the subsequent kinases RAF, MEK and extracellular response kinase 1 and 2 (ERK1 and 2) and promotes cell survival, proliferation, differentiation and apoptosis [152]. Various MAPK proteins have been linked to therapy resistance in cancer. The p38 MAPK promoted resistance to trastuzumab in breast cancer [153] and MEK and ERK promoted resistance to saracatinib in ovarian cancer [154]. A study in rabbit adipose derived stem cells, demonstrated there was a role of molecular weight of HA in activation of ERK, where increasing molecular weights of HA, 80 kD to 600 kDa to 2000 kDa increased activation of phospho-ERK [155]. Contradictory to this relationship LMW–HA (unspecified weight) and not ~1600 kDa HA activated the MAPK pathway in JEG-3 choriocarcinoma cells via RHAMM, indicating a possible receptor or cell type dependent effect of HA [77]. In SKOV-3 cells, 1000 kDa HA promoted CD44v3-Vav2 complex formation to activate Ras signaling [65]. Another study in ovarian cancer CaOV-3 and SKOV-3 cell lines, found EGF stimulated MAPK/ERK and mediated cell migration [156]. In CaOV-3 cells, this interaction was also dependent on HA and CD44 [156]. This co-operative relationship between EGF and HA was similarly observed in head and neck cancer cells (HSC-3) [73]. 500 kDa HA promoted the formation of a complex between EGFR and CD44 to activate Ras GTPase, Raf and ERK promoting cell invasion and migration [73,157]. Overall, there is limited evidence for the role of molecular weight of HA in activation of MAPK pathway in cancer. Activation of this pathway appears to be dependent on cell type and HA receptor interactions. Additionally, interaction of HA with CD44 can promote complex formation with other receptors which could additionally mediate effects of different HA molecular weights.

## 5. Targeting HA in Cancer

The role of HA in promoting tumor growth, progression and therapy resistance in cancers and its association with reduced survival suggest that HA is a worthy therapeutic target for treatment of cancer and enhancing current therapies. There are a several strategies that have been used for targeting HA in cancer including inhibition of HA synthesis, blocking HA signaling and promoting HA degradation. 4-Methylumbelliferone (4-MU) inhibits HA synthesis by sequestering glucuronic acid and decreasing expression of *HAS2* and *HAS3* [158]. It was initially administered as a dietary supplement however, it has been demonstrated to decrease proliferation, invasion, migration and in vivo tumor growth in prostate, breast, ovarian and melanoma carcinomas [159,160,161,162,163,164]. Additionally, treatment with 4-MU can enhance the effectiveness of other therapies as seen in treatment of fibrocarcinoma cells when used in combination with ionizing radiation [156] and combination with MEK1 inhibitor trametinib in malignant pleural mesothelioma [165]. Inhibition of PI3K can also reduce hyaluronan mediated effects on metastasis in pancreatic cancer [166]. Inhibition of HA-CD44 interactions have been investigated with anti-CD44 therapies, however high toxicity has been reported in clinical trials (reviewed in [167]). Administration of hyaluronidase, PEGPH20 to prostate cancer reduces *in vitro* adhesion and invasion and in vivo metastasis [24]. Lokeshwar and Selzer et al. hypothesized the function of HYAL was dependent on its concentration [168]. PEGPH20 initially degrades stromal HA before removing tumor associated HA, which contributes to breakdown of the stroma allowing access to chemotherapeutic drugs [169]. A recent clinical trial has reported improvements in progression free survival in patients treated with both PEGPH20 and nab-paclitaxel/gemcitabine compared to nab-paclitaxel /gemcitabine alone [170]. However, a more recent phase IB/II trial evaluating PEGPH20 together with mFOLIRINOX (oxaliplatin, leucovorin calcium, irinotecan hydrochloride & 5-fluorouracil) in patient with metastatic pancreatic cancer (NCT01959139) did not report a survival benefit for the combination treatment and found increased toxicity in patients receiving the PEGPH20 combination [171]. There are currently multiple clinical trials evaluating PEGPH20 in combination with a variety of chemotherapy drugs therapies (NCT02921022 NCT02487277, NCT02715804) including anti-PD-1 (NCT03481920) in pancreatic cancer and gall bladder cancer (NCT03267940). In future studies it will be important to investigate whether PEGPH20 treatment leads to increased levels of small HA polymers and whether these could have detrimental effects on patients. Given that different molecular weights of HA have different effects on cancer cells, developing methods to target specific HA molecular weights should also be considered in future.

## 6. Conclusions

HA, a key component of the ECM, plays numerous roles in the tumor microenvironment, activating signaling cascades in tumors and exhibiting multiple downstream pro-cancer effects (summarized in Figure 1 and Figure 2). HA plays a complex role in a number of cancers, promoting not only cell growth, but relapse and therapy resistance, contributing to poor survival rates. This review summarized the role of HA in cancer, focusing on chemotherapy resistance and exploring how its molecular weight is important in both normal and cancer cell biology. A molecular weight range of 200–500 kDa appears to be important in promoting cancer cell stemness, however, the understanding of the functional role of different HA molecular weight on HA signaling pathways in cancer cells is still unclear. The novel finding of tumor resistance by vHMW-HA in the naked mole rat provides a future direction in cancer prevention. Further research is required to better understand the molecular mechanisms by which different HA molecular weight promote cancer progression and chemoresistance and to aid the development of therapeutic targets to overcome chemotherapy resistance.

## Figures and Tables

**Table 1:** Range of studies analyzing physiological role of exogenous hyaluronan (HA) of known molecular weights.

Cell Type	HA (kDa)	Functional role and effects of HA	Reference
Inflammation			
Mouse knee chondrocytes	50, 1000, 5000	50 kDa promoted and 5000 kDa reduced inflammatory response	[41]
Nasal epithelium RPMI 256	370, 900, 1600	1600 kDa decreased the inflammatory effects and 900kDa reduced ROS production	[42]
BALB/c mice	250, 470, 780, 900, 1200	780, 900 and 1200 kDa reduced and 250 kDa increased liver injury. 900 kDa HA reduced inflammation	[40]
RAW 264.7 and MHS macrophages	11, 52, 87, 250, 970	No molecular weight of HA stimulated macrophage inflammatory response	[45]
Primary mesangial cells	1.5, 3	HA have no effect and HYAL (containing endotoxins) stimulated an inflammatory response	[46]
BMDM and BMDC	10, 28, 243, 1680 Rooster comb Umbilical cord	Only umbilical cord HA and HYAL stimulated BMDM and BMDC due to endotoxin contamination	[44]
J774A, ATCC, TIB-67 murine macrophages	oHA, 5, 60, 800, 3000	oHA, 5 and 60 kDa were pro-inflammatory, 800 and 3000 kDa anti-inflammatory	[48]
Peripheral blood mononuclear cells (PBMC)	~2000, ~80–800	2000 kDa HA promotes and 80–800 kDa HA inhibits the differentiation of PBMC cells to fibrocytes	[13]
Angiogenesis and Wound Repair			
BAEC (Bovine aortic endothelial cells)	oHA (3–16 disaccharides) Rooster comb HA	oHA promotes BAEC proliferation and angiogenensis, inhibited by rooster comb HA	[53]
BAEC	Rooster comb HA oHA	oHA via MAPK promotes BAEC proliferation and wound healing	[54]
BAEC	oHA (unspecified biological source)	oHA promotes and native HA inhibits BAEC proliferation and expression of angiogenesis early response genes	[55]
Porcine vascular endothelial cells	oHA (2–10 disaccharides) Umbilical cord HA	oHA promoted cell proliferation, wound healing and migration via MAPK and RHAMM. Native HA promoted cell migration	[56]
Human umbilical vein endothelial cells Sprague-Dawley	oHA (2–10 disaccharides)	oHA promotes cell proliferation, tube formation and in vivo and in vitro wound healing	[57]
Arterial Smooth Muscle Cells	2100	HA promotes cell migration via RhoA and Rac kinases, PI3K activates Rac	[60]
NIH-3T3 Mouse fibroblasts	980, 132, 31, 2.3	980 kDa HA promotes cell invasion via NFκB activity and Snail2 expression	[59]
Sprague Dawley rats dermal fibroblasts	5, 40, 500, oHA: 4, 6, 8 and 10 mer	6 mer & 8 mer oHA promoted wound healing in vivo & in vitro via CD44 and RHAMM, 6mer oHA recruits M1 and M2 macrophages	[58]
Human umbilical vein endothelial cells Sprague-Dawley rat	oHA (2–10 disaccharides)	oHA promotes in vivo and in vitro cell proliferation and wound healing	[57]
Embryogenesis and Gland Branching			
Primary Trophoblasts	HMW-HA MMW-HA LMW-HA (unspecified weights)	HMW-HA and MMW-HA promote cell proliferation, invasion and survival. LMW-HA has no effect	[61]
Mammary epithelial cell line (Ep-H4)	6–21, 50, 240, 500	240 kDa and 500 kDa HA inhibit and 6–25 kDa HA promote mammary gland branching	[62]
Holtzman rat kidneys	234.4, 132.3, 64, 17, 6.55	234.4 kDa and 132.3 kDa HA inhibit ureteric gland morphogenesis, 17 kDa and 6.5 kDa stimulates branching morphogenesis.	[63]

RPMI: Roswell Park Memorial Institute; ROS: reactive oxygen species; HYAL: hyaluronidase; BMDM: bone marrow derived macrophages; BMDC: bone marrow derived dendritic cells; PBMC: peripheral blood mononuclear cells; BAEC: bovine aortic endothelial cells; HMW: high molecular weight; LMW: low molecular weight.

**Table 2:** Range of studies analyzing effects of exogenous HA of known molecular weights on cancer cells.

Cell Type	HA (kDa)	Functional role and effects of HA	Reference
Ovarian cancer			
SKOV-3	1000	HA promotes cell migration and growth via Rac1 and Ras	[65]
SKOV-3	500	HA promotes cell migration via CDC42 and ERK1	[66]
SKOV-3 OV-90 OVCAR-3 OVCAR-5	Umbilical Cord HA oHA (6–10)	HA promotes resistance to carboplatin via *ABCB3*, *ABCC2*, *ABCC3* and *ABCC1* expression. oHA abrogated HA effect	[8]
SKOV-3	500	HA increases *REX1, SOX2* and *MDR1* expression, promoting drug resistance	[67]
Breast cancer			
MDA-MB-231	1000	HA promotes cell growth and invasion via RhoA	[68]
MCF-7	500	HA increases *REX1, SOX2* and *MDR1* expression, promoting drug resistance	[67]
MCF-7	500	HA promotes MDR1 and Bcl-x_L_ (anti-apoptotic) expression, cell growth and invasion	[69]
MDA-MB-231	400–500	HA promotes cell growth and invasion via RhoA, RhoC and ROK	[70]
MDA-MB-231	3–5 500–1000	3–5 kDa promotes cell invasion	[71]
4T-1 SKBR-3	35, 117	35 kDa promotes cell migration and invasion	[72]
Head and neck squamous cell carcinoma			
HSC-3	500 Rooster Comb	HA via CD44 complexes with leukemia associated RhoGEF (LARG) and epidermal growth factor receptor (EGFR). Promotes cell migration and growth	[73]
HSC-3	500	HA promotes cell migration, proliferation and cisplatin resistance via PI3K and ROK	[74]
HSC-3	5, 20, 200, 700	200 kDa HA promotes stemness and cisplatin resistance	[75]
HSC-3	500–700	HA promoted expression of CSC markers, sphere and clone formation, cell growth and invasion, cisplatin resistance via RhoC	[76]
Choriocarcinoma			
JEG-3	LMW-HA (unspecified weight) 1500–1800	LMW-HA promotes cell migration via RHAMM, PI3K and MAPK	[77]
Leukemia			
K562 Vincristine sensitive Kv562 Resistant	1500–1800 oHA	HA promotes cell proliferation via CD44, inhibited by 4-methylumbelliferone (4MU) and oHA (in K562)	[78]

Rac1: Ras-related C3 botulinum toxin substrate 1; Cdc42: cell division control protein 42; ERK: extracellular signal regulated kinase; PI3K: phosphoinositide 3-Kinase; RhoC: Ras homolog gene family, member C; RhoA: Ras homolog gene family, member A; ROK: Rho-associated protein kinase; RHAMM: receptor for hyaluronan mediated motility; MAPK: mitogen activated protein kinase.
